# Single Cell Expression Systems for the Production of Recombinant Proteins for Immunodiagnosis and Immunoprophylaxis of Toxoplasmosis

**DOI:** 10.3390/microorganisms12081731

**Published:** 2024-08-22

**Authors:** Karolina Sołowińska, Lucyna Holec-Gąsior

**Affiliations:** Department of Biotechnology and Microbiology, Faculty of Chemistry, Gdańsk University of Technology, 11/12 Narutowicza Str., 80-233 Gdańsk, Poland; karolina.solowinska@pg.edu.pl

**Keywords:** *Toxoplasma gondii*, toxoplasmosis, recombinant antigen, expression systems, *Escherichia coli*, *Pichia pastoris*, *Leishmania tarentolae*, serological detection, vaccine development

## Abstract

Toxoplasmosis represents a significant public health and veterinary concern due to its widespread distribution, zoonotic transmission, and potential for severe health impacts in susceptible individuals and animal populations. The ability to design and produce recombinant proteins with precise antigenic properties is fundamental, as they serve as tools for accurate disease detection and effective immunization strategies, contributing to improved healthcare outcomes and disease control. Most commonly, a prokaryotic expression system is employed for the production of both single antigens and multi-epitope chimeric proteins; however, the cloning strategies, bacterial strain, vector, and expression conditions vary. Moreover, literature reports show the use of alternative microbial systems such as yeast or *Leishmania tarentolae*. This review provides an overview of the methods and strategies employed for the production of recombinant *Toxoplasma gondii* antigenic proteins for the serological detection of *T. gondii* infection and vaccine development.

## 1. Introduction

Toxoplasmosis caused by the intracellular, protozoan parasite *Toxoplasma gondii* (*T. gondii)* is one of the most common infections worldwide. It has been estimated that around one third of the human population has been infected by the parasite. However, seroprevalence rates can vary from as low as 0.5% to over 80%, depending on climate, dietary practices, social customs, hygiene standards, and urban development [1]. Many possible hosts (i.e., birds and mammals, including humans) as well as multiple routes of transmission (discussed below) contribute to the widespread distribution of *T. gondii* [2].

Toxoplasmosis presents a range of nonspecific symptoms. The majority of infected individuals are asymptomatic or exhibit mild flu-like symptoms; however, for those with weakened immune systems, such as HIV/AIDS patients, organ transplant recipients, or cancer patients undergoing certain types of chemotherapy, toxoplasmosis can be severe and potentially life threatening [3]. Infection can be acquired either congenitally or postnatally. For congenital transmission, the risk and severity are dependent on the stage of pregnancy at the time of infection. While infection early in pregnancy carries a lower risk of transmission, it can lead to much more severe outcomes if the fetus does become infected, including miscarriage, stillbirth, or serious developmental disorders. As gestational age increases, so does the risk of transplacental transmission; however, the severity of the disease significantly decreases [4].

Several pathways for postnatal infection have been identified, including mainly the consumption of undercooked meat containing *T. gondii* tissue cysts. Furthermore, infection can occur by ingesting food or water contaminated with oocysts, which are shed in the feces of infected cats-definitive hosts of the parasite. Additionally, accidental ingestion of these oocysts after handling cat litter or soil that has been contaminated with feces from infected cats can lead to infection. Rarely, transmission can take place by organ transplantation or blood transfusion [5].

*T. gondii* exhibits a significant antigenic complexity, which is likely related to its ability to infect and persist in a wide range of hosts. This diversity is a result of three different infective life forms (tachyzoites, bradyzoites, and sporozoites), each presenting unique sets of antigens that interact with the host immune system. These include surface antigens (SAGs), apical membrane antigens (AMAs) and secretory proteins, such as dense granules (GRAs), rhoptry proteins (ROPs), matrix antigens (MAGs) and micronemes (MICs) [6], all of which have become popular targets for immunodiagnostics and vaccine development.

The accurate diagnosis of toxoplasmosis is imperative for monitoring, prevention, and management of this parasitic disease. Traditional diagnostic methods rely on serological techniques that detect anti-*T. gondii* antibodies in serum samples. Recombinant proteins, which can be produced consistently and in large quantities, offer a reliable source of antigens that improve the specificity and sensitivity of these diagnostic assays [7,8]. Several commercial kits have been developed for the diagnosis of toxoplasmosis, e.g., enzyme immunoassay (EIA), *Toxoplasma* immunoglobulin G (IgG) (TestLine Clinical Diagnostics), Human *Toxoplasma gondii* IgG Rapid Test Kit (Abbexa), Human Anti-*Toxoplasma gondii* immunoglobulin M (IgM) enzyme-linked immunosorbent assay (ELISA) Kit (Abcam), Human Anti-*Toxoplasma gondii* IgG ELISA Kit (Abcam), Human *Toxoplasma* (TOX) IgM ELISA Kit (Elabscience). However, most are based on whole tachyzoite antigens, while the use of recombinant proteins is still limited. Furthermore, the absence of an effective human vaccine and the limited use of available animal vaccines highlight the urgent need for continued research in this area. The only registered vaccine, containing live attenuated *T. gondii* S48 strain tachyzoites, reduces the incidence of abortions and neonatal mortality from congenital infections in sheep but does not offer broader protection against the disease [9,10]. Recombinant proteins are central to vaccine development efforts because they can be engineered to enhance immune responses and are safer than using live or attenuated parasites [11,12].

Choosing the right expression system for the production of *Toxoplasma* antigens impacts the effectiveness and applicability of the resulting antigens in many ways. Firstly, eukaryotic *T. gondii* proteins often require specific post-translational modifications (PTMs), which can directly and indirectly influence protein immunogenicity, as the modification can in itself trigger an immune response, or its presence can affect protein folding [13]. Epitopes can be broadly categorized into two main types: linear (sequential) epitopes, which are recognized by antibodies based on their primary amino acid sequence, regardless of the protein’s folded structure, and conformational (discontinuous) epitopes that are formed by the three-dimensional folding of the protein, bringing together amino acids that are distant in the linear sequence but close in the folded structure. They are recognized by antibodies based on the shape and the spatial arrangement of these amino acids. It is also known that approximately 90% of B-cell epitopes are conformational [14]. This clearly shows that changes in PTMs affecting protein folding and the subsequent three-dimensional structure can alter epitope presentation, leading to varying immunogenicity depending on the chosen expression system. Furthermore, the possible harmful by-products or contaminants are dependent on the chosen expression system. Any impurities may impact the biological safety of produced recombinant proteins, which is especially important in vaccine development. Lastly, choosing a system for the production of heterologous proteins must take into consideration costs. Mammalian cell lines are significantly more expensive and laborious in comparison to microbial systems. Moreover, factors such as purification complexity also impact production costs.

**Figure 1:**
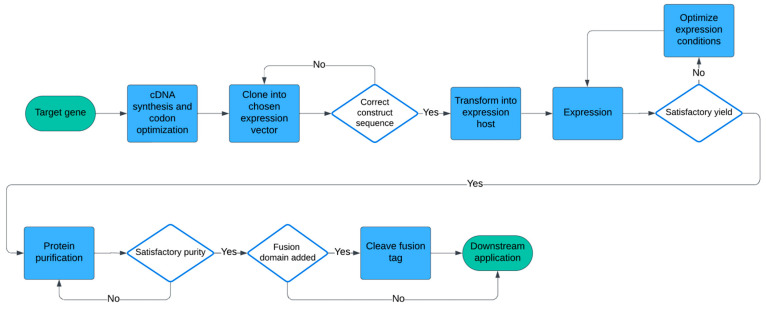
Flowchart illustrating the process of heterologous protein expression in microbial systems.

This paper aims to explore microbial expression systems currently employed in the production of recombinant proteins for the immunodiagnosis and immunoprophylaxis of toxoplasmosis, examining their benefits and limitations. Through this analysis, we seek to highlight how the expression system and production strategy influence the immunogenicity of *Toxoplasma* antigens and therefore affect the efficacy and reliability of toxoplasmosis management strategies. A general workflow of producing heterologous proteins in microbial expression systems is shown in Figure 1.

## 2. Materials and Methods

The search for articles containing information regarding the biotechnological production of *T. gondii* recombinant proteins, in order to extract information on the employed expression systems, was conducted in the following electronic databases: PubMed, Scopus and Google Scholar. The following research strings and Boolean operators have been entered in each database: (“toxoplasma gondii” OR “T. gondii” OR “toxoplasmosis”) AND (“diagnosis” OR “vaccine” OR “recombinant protein” OR “expression system”). Manual searches of the reference lists of the included articles to identify other potential sources were also carried out. The search was performed without date restrictions. Titles and abstracts were screened by both authors independently. The full text of relevant papers was reviewed, and any disagreement on articles selected was resolved.

Studies were included if they satisfied all the following criteria: (1) published in English; (2) focused on *T. gondii* recombinant proteins with diagnostic and/or immunoprophylactic applications; (3) if diagnostic assays were conducted, human serum samples were used; (4) protein expression was carried out in a unicellular, microbial organism; (5) detailed information regarding the construction of expression system was available.

After reviewing all articles, papers without sufficient information and that did not meet the minimum criteria were excluded. From each eligible study, information regarding publishing year, cloning strategy, expression vector, host organism, expression conditions, production yield, ability of produced protein to recognize anti-*T. gondii* antibodies, the sensitivity and specificity of serodiagnostic assays based on recombinant protein, and/or the immune response and survival times of mice vaccinated with recombinant protein was collected.

## 3. Prokaryotic Expression Systems

The production of heterologous proteins is predominantly carried out in bacterial expression systems. The rapid growth, low-cost media, ease of genetic manipulation, and well-characterized genetics of prokaryotic expression platforms has contributed to advancing the study of recombinant *T. gondii* antigenic proteins [15]. Many bacteria have shown promise as expression systems. These include *Lactoccocus lactis* (*L. lactis*), *Pseudomonas* species, *Streptomyces* systems, Coryneform bacteria [16], and *Bacillus* strains [15]. However, all studies on the production of *Toxoplasma* antigenic proteins prokaryotes employ *Escherichia coli* (*E. coli*). On account of the increasing availability of various cloning vectors and mutant host strains, each offering unique advantages for different applications or challenges, recombinant *T. gondii* protein production in *E. coli* cells is characterized by a diversity of methods rather than a one-size-fits-all approach. Studies report on variable host strains, vectors, and expression conditions for the production of single and chimeric recombinant *Toxoplasma* antigens.

### 3.1. E. coli Expression Systems

The cornerstone of recombinant protein expression in *E. coli* is the *lac* promoter, which is a key element of the *lac* operon. Genes are expressed in the presence of lactose or synthetic lactose analogs and the absence of easily metabolizable carbon sources, such as glucose, found in rich media. To mitigate this catabolite repression, the *lac*UV5 promoter was introduced [17]. However, both the *lac* and *lac*UV5 promoters maintain a basal level of uninduced expression (leakiness) when present in multicopy plasmids due to the low level of the chromosomally coded LacI repressor [18]. Efficient repression requires the use of a *lacIq* promoter, present for example in the NEBExpress Iq strain (New England Biolabs), which significantly increases LacI expression. Alternatively, basal transcription from the *lac*UV5 promoter can be decreased by supplementing media with glucose [19].

The most notable vectors utilizing the lac promoters are the pUC series (*lac*UV5 promoter) commercialized by Thermo Scientific [17]. While pUC plasmids can technically be used for protein production, their design, features, and very high copy number make them ideal for cloning and DNA manipulation tasks. Many studies on the production of *T. gondii* recombinant proteins utilize pUC plasmids as cloning vectors and subsequently subclone the gene of interest into other plasmids optimized for tightly regulated, high-level protein expression with additional features such as tags for purification and detection. In 1983, De Boer at al. [20] described the *tac* promoter, which was created by fusing the −35 region of the *trp* promoter with the −10 region of the *lac* promoter. This hybrid is not only significantly stronger but also provides a tight regulation of gene expression inducible by lactose or IPTG (isopropyl β-D-1-thiogalactopyranoside) and is used in the pMAL (N-terminal MBP tag, New England Biolabs) or pGEX (N-terminal GST tag, Cytivia) series vectors. Studies reporting the use of pMAL plasmids for the production of *T. gondii* recombinant proteins are limited and mostly focused on the identification and characterization of proteins, their kinetic parameters, and role in parasite invasion [21,22,23,24,25,26,27,28,29], while pGEX plasmids have been used for the production of immunogenic *Toxoplasma* proteins assessed in regard to their immunodiagnostic and/or immunoprophylactic potential (Table 1). This difference can be most likely attributed to their distinct N-terminal fusion tags, both allowing protein detection, purification, increased expression, and increased solubility. The 26 kDa glutathione *S*-transferase (GST) tag enables protein purification on commercially available, relatively reusable resin by affinity chromatography in low concentrations of nonionic detergents as well as denaturing and reducing agents. Mild elution conditions preserve protein antigenicity, and GST can be conveniently cleaved from the protein of interest while still bound to the glutathione resin [30]. Moreover, this tag can protect the target protein against intracellular proteases of the expression host [31]. In contrast, the maltose binding protein (MBP) is larger (45 kDa) and can therefore have a stronger impact on protein properties. Purification of MBP fusion proteins by affinity chromatography must be performed in the absence of denaturing and reducing agents. Furthermore, the proteolytic removal of an MBP tag while the fusion protein is bound to resin is not possible [30]. The use of GST fusion proteins in diagnostic assays without cleaving the added domain has been analyzed. Tenter et al. [32] stated that the cross-reactivity of GST-labeled antigens depends on the concentration of fusion protein and serum dilution. Parmley et al. [33] included GST as a control antigen in immunoblots and ELISA. The authors observed a background reaction to GST in some sera; however, the reactivity was noticeably weaker than the reactivity of a recombinant GST-P22 protein. Similarly, Redlich et al. [34] included a GST-IgG control and found that only 4% (7/159) of human serum samples reacted positively with glutathione S-transferase. Additionally, a 2000 study [35] found that GST-labeled protein showed no reactivity in an IgG ELISA. Wang et al. [36] immunized mice with both GST-ROP17 or a GST control. Following challenge, it was determined that the survival rates of mice immunized with rROP17 were significantly increased (75%) when compared to those of the GST-treated control mice. These examples suggest that the impact of glutathione S-transferase on recombinant antigen immunogenicity is negligible; however, the cleavage of fusion domains increases the specificity of both vaccines and diagnostic assays.

#### T7 System

The T7 promoter system present in pET vectors (Novagen) is undoubtably the system of choice for recombinant protein production in *E. coli*. Target genes are cloned downstream of a strong bacteriophage T7 promoter and transformed into bacterial strains which carry the T7 RNA polymerase (RNAP) gene under the control of the inducible *lac*UV5 promoter. Upon induction, the highly active T7 RNAP effectively outcompetes the host’s RNA polymerase for transcriptional control. Subsequently, the target protein can constitute over 50% of the cellular protein within just a few hours, and expression levels can be lowered by decreasing the inducer concentration. Initially recombinant plasmid stability can be established by cloning target genes into *E. coli* strains lacking the T7 RNAP gene, which ensures the cloned genes remain transcriptionally silent until induction. Stable plasmids can then be transferred to host cells coding T7 RNAP, such as BL21(DE3). Basal expression from the leaky *lac*UV5 promoter can be minimized by employing *E. coli* host strains that carry a plasmid coding for T7 lysozyme expressed at low levels, which binds to T7 RNAP and inhibits uninduced transcription. These are known as pLysS and pLysE hosts. *Toxoplasma* antigenic proteins expressed in the T7 system are summarized in Table 2.

Novagen offers a multitude of pET plasmids carrying different fusion partners, e.g., His-tag, S-tag, T7-tag, GST-tag, Nus-tag or HSV-tag. Among them, polyhistidine tags are most commonly used. Their main advantages include their small size (∼2.5 kDa) and the possibility of protein purification under both native and denaturing conditions. What is more, the matrix can be indefinitely regenerated and reused [30]. The effects of His-tags on protein structure, solubility and immunogenicity are ambiguous. It is generally believed that due to their small size, their influence is insignificant, and many studies support this hypothesis, showing that the addition of His-tags has no influence on protein structure [44] or the induction of immune responses [45,46]. Conversely, researchers reported that the presence of polyhistidine domains does affect antibody–antigen binding [47] and alters humoral and cellular immune responses [48]. These inconclusive findings highlight the importance of determining the impact of histidine tags on protein properties on a case-by-case basis—an overlooked approach in the reviewed literature.

**Table 2:** *T. gondii* antigens produced in *E. coli* T7 system.

Plasmid Vector	*E. coli* Host Strain	Protein	Expression Conditions	Yield	Application		Results	Reference, Year
					Diagnostic	Vaccine		
pET29	BL21 (DE3) pLysS	S-B10^a^-6xHis	37 °C, 3 h	−	+	−	Strong immunoreactivity with human sera from both chronic and acute infections.	1998, [49]
pUET1	Rosetta (DE3) pLysS	6xHis-GRA6_30–231_-6xHis 6xHis-P35^b^_26–170_-6xHis 6xHis-SAG2_30–170_-6xHis	LB, 30 °C, 8 h LB, 30 °C, 8 h LB, 37 °C, 3 h	60–80 mg/L induced bacterial culture	+	−	Both r-GRA6 and r-p35 antigens detected antibodies more frequently (*p* < 0.01) from acute (93.9 and 87.9%) rather than chronic (60.6 and 53.0%) infections. The r-SAG2 gave a similar sensitivity in both groups of patients (93.9 and 95.5%) Both r-GRA6 and r-p35 antigens detected antibodies more frequently (*p* < 0.01) from acute (93.9 and 87.9%) rather than chronic (60.6 and 53.0%) infections. The r-SAG2 gave a similar sensitivity in both groups of patients (93.9 and 95.5%) GRA6 and p35 detected antibodies more frequently in acute infections (93.9% and 87.9%) compared to chronic infections (60.6% and 53.0%). SAG2 showed similar sensitivity in both acute and chronic cases (93.9% and 95.5%).	2005, [50]
pET32a(+)	BL21 (DE3)	TRX-(Hisx6)-GRA2	LB, 37 °C, 3 h	12 mg/L induced bacterial culture	+	−	Specificity of IgG ELISA was 96.4%. Sensitivity was 95.8% to 100% for acute infection sera and 65.7% to 71.4% for chronic infection sera.	2007, [51]
pUET1	Rosetta (DE3) pLysS	6xHis-MAG1_30–222_-6xHis	LB, 37 °C, 16 h	90 mg/L induced bacterial culture	+	−	Can distinguish between acute (97.3% sensitivity) and chronic (7.5% sensitivity) phases of toxoplasmosis.	2007, [52]
pUET1	Rosetta (DE3) pLysS	6xHis-MIC1_25–182_-6xHis 6xHis-MIC1_183–456_-6xHis 6xHis-MIC1_25–456_-6xHis	LB, 37 °C	16–24 mg/L induced bacterial culture	+	−	The three recombinant MIC1 proteins showed similar antigenicity for acute toxoplasmosis sera, but chronic infection sera had significantly lower sensitivity.	2008, [53]
pET-28b(+)	Rosetta (DE3)	6xHis-GRA7_18–236_-6xHis	LB, 37 °C, 5 h	−	+	−	Western blot results indicated strong recognition of GRA7 by acute sera, weak detection by chronic sera, and no specific bands in negative sera.	2009, [54]
pUET1	Rosetta (DE3) pLysS	6xHis-ROP1_85–396_-6xHis 6xHis-GRA2_24–185_-6xHis	LB, 37 °C, 16 h	16 mg/L 28 mg/L induced bacterial culture	+	−	GRA2 and ROP1 showed higher sensitivity in acute infection sera (100% and 94.6%) than in chronic infection sera (22.5% and 15.5%).	2009, [55]
pUET1	BL21 (DE3) pLysS	6xHis-GRA5_26–120_-6xHis	LB, 30 °C, 8 h	15 mg/L induced bacterial culture	+	−	Anti-GRA5 IgG antibodies were found in 70.9% of seropositive samples, similar to TLA-ELISA results.	2010, [56]
pET-28b(+)	Rosetta (DE3)	6xHis-S-GRA8_23–169_-6xHis	LB, 30 °C, 5 h	68 mg/L induced bacterial culture	+	−	IgM GRA8-ELISA had 97.1% specificity and 60.6% sensitivity.	2011, [57]
pET-32c	BL21 (DE3)	Trx-(Hisx6)-SAG1_309–318_-SAG2_109–118_-SAG3_347–356_-6xHis	LB, 37 °C, 4 h	−	+	−	IgG ELISA showed 94.4% sensitivity and 100% specificity. IgM ELISA showed 96.9% sensitivity and 100% specificity	2012, [58]
pUET1	Rosetta (DE3) pLacI	6xHis-MIC1_25–182_-MAG1_30–222_-6xHis	LB, 37 °C, 16 h	43 mg/L induced bacterial culture	+	−	The IgG MIC1-MAG1-ELISA showed 90.8% sensitivity, similar to TLA (91.8%) and higher than MIC1, MAG1, or their mixture.	2012, [59]
pUET1	-	6xHis-MIC1_25–182_-MAG1_30–222_-SAG1_49–198_-6xHis	−	20 mg/L induced bacterial culture	+	−	The MIC1-MAG1-SAG1-ELISA had 100% specificity and nearly 100% sensitivity, outperforming assays with just MIC1-MAG1 protein.	2012, [60]
pET-30a(+)	Rosetta (DE3)	6xHis-PDI^c^-6xHis	25 °C, 8 h	−	−	+	Nasal immunization with PDI induced a protective immune response in mice, increasing the 30-day survival rate by about 31% compared to the control group.	2013, [61]
pET-32a	BL21 (DE3) pLysS	6xHis-GRA4-6xHis	LB, 6–8 h	−	−	+	Subcutaneous immunization induced high IgG levels, which declined after one week, and elevated IFN-γ, interleukin (IL) 10, and IL-4. However, it did not confer protection against *T. gondii* in mice.	2013, [62]
pET-30 Ek/LIC	Rosetta (DE3) pLacI	6xHis-SAG2-GRA1-ROP1_85–396_-6xHis 6xHis-SAG2-GRA1-ROP1_85–250_-6xHis	LB, 30 °C, 18 h	31 mg/L 33 mg/L induced bacterial culture	+	−	IgG ELISA using SAG2-GRA1-ROP1_85–396_—100%, sensitivity. IgG ELISA using SAG2-GRA1-ROP1_85–250_—88.4% sensitivity.	2015, [63]
pUET1	Rosetta (DE3) pLacI	6xHis-P35_26–170_-MAG1_30–222_-6xHis 6xHis-MIC1_25–182_-ROP1_113–295_-6xHis 6xHis-MAG1_30–222_-ROP1_113–295_-6xHis	LB, 37 °C, 16 h	43 mg/L 25 mg/L 36 mg/L induced bacterial culture	+	−	The reactivity of IgG ELISA for acute toxoplasmosis sera was 100% for P35-MAG1, 77.3% for MIC1-ROP1, and 86.4% for MAG1-ROP1, significantly higher than for chronic infection sera (26.2%, 36.1%, and 32.8%, respectively). IgM ELISA using P35-MAG1, MIC1-ROP1, and MAG1-ROP1 had a sensitivity of 81.8%, 72.7%, and 59.1%, respectively	2015, [64]
pET-30a(+)	BL21 (DE3)	6xHis-ADF^d^-6xHis	LB, 25 °C, 12 h	−	−	+	Intranasal immunization increased secretory IgA, IgG titers, splenocyte proliferation, and IL-2 and IFN-γ secretion. It improved survival by 36.36% and reduced liver and brain tachyzoite loads by 67.77% and 51.01%.	2016, [65]
pET-28α	BL21 (DE3)	6xHis-ROP18-6xHis	LB, 30 °C, 6 h	−	−	+	Intranasal immunizations with ROP18 in nanospheres induced higher levels of IgA and IgG2a as compared to groups inoculated intranasally with ROP18 alone or subcutaneously injected with ROP18 in montanide adjuvant.	2017, [66]
pET-28α	BL21 (DE3)	6xHis-SAG1-6xHis	LB, 30 °C	−	−	+	Intranasal immunization with SAG1 in nanospheres induced higher specific IgA and IgG2a responses compared to controls.	2018, [67]
pET28a	BL21	6xHis-CDPK3^e^-6xHis	LB, 30 °C, 6 h	−	−	+	Intramuscular immunization induced spleen cell proliferation, IFN-γ release, and high IgG titers. It partially protected against acute toxoplasmosis, reducing brain cysts by 46.5%.	2019, [68]
pET28a	BL21 (DE3)	6xHis-GRA2-6xHis 6xHis-GRA7-6xHis 6xHis-TPI^f^-6xHis	LB, 37 °C, 4 h	−	+	−	The sensitivities of GRA2, GRA7, TPI, and their mixture were 85%, 83.3%, 88.3%, and 96.7%, respectively, with specificities of 85%, 90%, 100%, and 100%. GRA2 and GRA7 showed cross-reactivity.	2019, [69]
pET-30 Ek/LIC	Rosetta (DE3) pLacI	6xHis -SAG2-GRA1-ROP1-AMA1_67–287-_6xHis 6xHis-AMA1_67–287_-SAG2-GRA1-ROP1-6xHis 6xHis-AMA1_287–569_-SAG2-GRA1-ROP1-6xHis 6xHis-AMA1_67–569_-SAG2-GRA1-ROP1-6xHis	TB, 23 °C, 18 h	11–23 mg/L induced bacterial culture	+	−	All chimeric proteins demonstrated 100% sensitivity and specificity in IgG ELISA. Avidity results were comparable to commercial assays.	2019, [70]
pET-30 Ek/LIC	Rosetta (DE3) pLysS	6xHis-AMA1_67–287_-6xHis 6xHis-AMA1_287–569_-6xHis 6xHis-AMA1_67–569_-6xHis	LB, 30 °C, 4 h	33 mg/L 31 mg/L 15 mg/L induced bacterial culture	+	−	The full-length AMA1 antigen outperforms its fragments in diagnostic assays. High reactivity with anti-*T. gondii* IgG (99.4%) and IgM (80.0%) antibodies.	2020, [71]
pET-28a	BL21 (DE3)	6xHis-MIC3_30–180_-ROP8_85–185_-SAG1_85–235-_6xHis	LB, 37 °C, 6 h	−	−	+	Mice immunized with MIC3-ROP8-SAG1 showed stronger humoral and Th1 responses. Co-immunization with Freund and calcium phosphate nanoparticles impaired responses. Survival time increased by 15 days.	2021, [72]
pET-28a (+)	BL21 (DE3)	6xHis-ROP18_377–546_—MIC4_302–471_–SAG1_130–299_-6xHis	LB, 37 °C, 6 h	−	−	+	Vaccinated mice, particularly with ROP18-MIC4-SAG1-Freund, showed high levels of total IgG, IgG2a, and IFN-γ. Survival time increased by 15 days after the challenge.	2023, [73]

^a^ a member of the excreted–secreted antigens (ESA) of *T. gondii*. ^b^ GRA8 (P35). ^c^ Protein disulfide isomerase (PDI). ^d^ Actin depolymerizing factor (ADF). ^e^ Calcium-dependent protein kinase 3 (CDPK3). ^f^ Triosephosphate isomerase (TPI).

Other well-known *E. coli* expression systems include the positively controlled, arabinose-induced araBAD (pBAD vectors, Thermo Scientific) or the tryptophan-inducible P_L_ expression system based on a promoter from bacteriophage lambda (pLEX vectors, (Thermo Scientific) [17]. To the best of our knowledge, no studies report the use of these systems for the production of *T. gondii* antigens. In 2004, Qing et al. [74] developed a series of cold shock-inducible expression vectors (pCOLDs), which are currently commercially available from Takara Bio. The *cspA* promoter is located downstream of the *lac* operator sequence, which minimizes basal expression at 37 °C. The addition of IPTG coupled with a temperature shift to 15 °C induces the expression of target genes which may be beneficial as the overexpression of heterologous genes in *E. coli* at low temperatures improves protein solubility and decreases proteolytic degradation by suppressing the expression of host proteins [75]. Sonaimuthu et al. [76] cloned the ROP8 gene into a pCOLD I DNA vector and produced a soluble his-tagged protein in BL21(DE3) host cells, which was followed by purification by metal affinity chromatography, yielding low amounts of pure protein. The expressed ROP8 protein was serodiagnostically evaluated by Western blot analysis with human serum samples showing 90% sensitivity and 94% specificity. A 2016 study [77] demonstrated the expression of a soluble 6x-His-ROP1 antigen in *E. coli* BL21(DE3) by cloning the target gene into a pCOLD plasmid. The authors found that strong and specific immunity is induced by the recombinant ROP1 in mice leading to partial protection against *T. gondii.* These results cannot clearly indicate if cold shock-inducible systems improve protein solubility, as a 2009 study also reported the production of soluble his-tagged ROP1 in *E. coli* with the use of a T7 system [55].

### 3.2. Protein Solubility

The overexpression of recombinant proteins in *E. coli* can result in their aggregation into insoluble inclusion bodies in the cytoplasm [78]. This is unfavorable, as incomplete or incorrect folding can inhibit both the formation of conformational epitopes and the exposure of linear epitopes [79]. Two strategies can be employed to circumvent this issue: (1) taking steps to prevent the formation of aggregates or (2) solubilization and refolding of target proteins from inclusion bodies in vitro.

Often, the challenge of achieving native protein conformations increases with the number of cysteine residues due to the various possible isoforms and complexity of disulfide bond patterns [80]. The correct folding of recombinant eukaryotic proteins in *E. coli* systems is limited by the reducing cytoplasmic environment maintained by several cellular components, including the thioredoxin and glutaredoxin systems, which are effective in reducing disulfide bonds back to free thiols [81]. One approach to mitigate this issue focuses on using strains engineered to promote an oxidative environment in the cytoplasm by knocking out thioredoxin reductase and glutaredoxin reductase (encoded by the genes trxB and gor, respectively) [82]. These Δ*gor* Δ*trxB* strains can be purchased commercially under the names Origami, Rosetta-gami (Novagen) or SHuffle (New England Biolabs). The SHuffle system additionally produces DsbC in the cytoplasm, which acts as a catalyst for disulfide bond isomerization [83]. Interestingly, Nguyen et al. [83] reported the production of eukaryotic proteins containing disulfide bonds in bacterial cells without disrupting the reducing pathways. The authors state that the co-expression of a sulfhydryl oxidase and a disulfide isomerase, catalysts of S-S bond formation, allows for the correct folding of proteins in the cytoplasm. A study published in 2020 described the use of this co-expression-based system in an *E. coli* strain with *gor* and *trxB* gene deletions for the production of two of the most widely studied *Toxoplasma* antigens—SAG1 and SAG2, in soluble, correctly folded forms [84]. The SAG1 antigen is of particular interest due to the presence of 6 disulfide bonds [85], three of which directly affect the formation of the dominant epitope [84].

Alternatively, expression of the recombinant protein may be targeted to the bacterial periplasm, where the environment is more oxidizing, naturally facilitating the formation of disulfide bonds. This can be achieved by using suitable expression vectors carrying signal peptides [86]. However, secreting proteins to the periplasm frequently results in low protein yields, which is likely due to the restricted volume of the periplasm and the limited capacity of the translocation machinery [80].

Moreover, most expression plasmids provide fusion partners that aid in protein solubilization, e.g., maltose binding protein (MBP), glutathione-S-transferase (GST), N utilization substance A (NusA), thioredoxin (TRX) [82], double Z-domain from staphylococcal protein A (ZZ), or Gb1-domain from protein G (Gb1) [80]. By enhancing solubility and stability, these tags can significantly increase the yield of functional protein; however, they must usually be cleaved from the target protein to maintain antibody–antigen interactions. Additionally, solubility does not guarantee correct folding, as fusion tags may keep a misfolded protein in a soluble state [87]. One study documented the expression of an immunogenic fragment of the *T. gondii* rhoptry protein ROP2, fused with TRX or MBP, which resulted in higher expression levels and more efficient renaturation compared to the untagged rROP2_196–561_. Furthermore, the fusion partners did not compromise the immunogenicity of the ROP2 fragment in ELISA with sera from *T. gondii*-seropositive individuals [88]. Similarly, Golkar et al. [51] reported that the addition of a TRX domain to GRA2 increases the expression level and solubility. The authors also developed an IgG ELISA using TRX-(Hisx6)-GRA2 as the coating antigen, further proving that the cleavage of solubility fusion tags is not necessary for serodiagnostic applications. On the other hand, Klein et al. [84] reported that N-terminal MBP influenced the binding of human antibodies to SAG1 as shown by lower levels of fluorescence (30–35%) in bead-based multiplex assays. The observed difference is most likely due to the major epitope of SAG1 present at the N-terminus [89]. Therefore, protein tags could hinder antibody access. These studies lead to the conclusion that the impact of fusion tags on antigen–antibody interactions is dependent on the localization of immunogenic epitopes.

As previously mentioned, the target protein may be produced as inclusion bodies and then refolded by physicochemical methods. Allahyari et al. [87] investigated the refolding of the SAG1 recombinant protein using three distinct techniques: dialysis in the presence of reduced/oxidized glutathione, drop-wise dilution and drop-wise dilution in the presence of CuSO_4_. They found that SAG1, when refolded via a two-step dialysis method with reduced/oxidized glutathione as the oxido-shuffling agent, exhibited significantly higher reactivity with anti-*Toxoplasma* IgG antibodies compared to the other two refolding approaches. Similarly, Mirzadeh et al. [90] performed the refolding of a SAG1-related sequence 3 (SRS3) protein by three methods and found that dialysis was the most favorable. The addition of glycerol to purified protein preparation to minimize the insolubility of *T. gondii* antigens after freeze–thaw cycles has been reported [91]. Notably, glycerol did not impact the diagnostic value in an IgG ELISA.

It is important to note that the production of recombinant proteins intended for therapeutic applications in *E. coli* is burdened by the presence of endotoxins, specifically lipopolysaccharides (LPSs) that can provoke strong immune responses in humans and animals, complicating the safety and efficacy of therapeutic proteins [92]. Techniques for endotoxin removal must be taken into consideration when choosing an *E. coli* expression system for the production of biopharmaceuticals. However, the most significant limitation of prokaryotic expression systems is their inability to carry out PTMs. In order to overcome this disadvantage, alternative expressions systems have been developed for the production of recombinant *T. gondii* proteins.

## 4. Yeast Expression Systems

Yeast offers a middle ground between the simplicity and cost-effectiveness of bacterial systems and the complex PTM capabilities of mammalian systems, although the glycosylation patterns are somewhat different. Moreover, yeast is capable of homologous recombination, allowing for the integration of linear foreign DNA into its genome, facilitating the creation of stable cell lines. Unlike some other expression systems, yeast cells can secrete the recombinant protein directly into the culture medium, which not only reduces the risk of product degradation and contamination but also simplifies the recovery and purification of the target protein. What is more, expression vectors can be readily engineered to allow for the expression of multiple copies of a target protein, multimeric proteins with different subunit structures, or proteins alongside their cognate binding partners [93,94,95]. The two most commonly used species are *Saccharomyces cerevisiae (S. cerevisiae)* and *Pichia pastoris (P. pastoris).* Although a 2018 study [96] reported the expression of *Toxoplasma* protein MIC16 on the surface of *S. cerevisiae* and examined its immunoprophylactic potential, *P. pastoris* has become the preferred host cell for extracellular production due to its capability to produce extremely high-density cultures in simple and inexpensive media as well as a more favorable glycosylation pattern [97]. *S. cerevisiae* is known to hyperglycosylate proteins, leading to glycans that are significantly larger and more branched than those in *P. pastoris*, which can affect protein binding, activity, and potentially yield an altered immunogenic response [97]. An additional strength of the *P. pastoris* expression system lies in the availability of strong and tightly regulated promoters that can drive the high-level expression of foreign genes, making it possible to produce large quantities of target proteins relatively easily [93]. Various other yeast strains, such as *Yarrowia lipolytica* (*Y. lipolytica*), *Arxula adeninivorans* (*A. adeninivorans*), and *Kluyveromyces lactis* (*K. lactis*), have been investigated as potential expression systems for vaccines and immunotherapeutic and biotherapeutic molecules [98]. However, there are no reported studies on the production of *T. gondii* antigens using these systems.

Efforts to enhance diagnostic tests for toxoplasmosis and to develop a preventive vaccine have focused significantly on the previously mentioned *T. gondii* surface antigen SAG1, which is a stable, non-variant antigen, which is conserved both immunologically and in its amino acid sequence [99]. Native SAG1 is a conformational antigen anchored in the parasite’s membrane via a glycosylphosphatidylinositol (GPI) group. Although it has a potential N-linked glycosylation site, studies have shown that this site is not functional in tachyzoites, despite the presence of necessary biological mechanisms [100]. Biemans et al. [101] reported the use of a *P. pastoris* system for the expression of a SAG1 antigen deleted from its C-terminal GPI-anchor, as authors the hypothesized that retaining the GPI region would inhibit the secretion of the protein by yeasts. The produced protein was secreted in a conformation suitable for recognition by monoclonal antibodies specific for native SAG1, indicating successful disulfide bond formation. It was shown that the potential N-glycosylation site was glycosylated, but it did not notably impact the conformation of the recombinant product. The yeast-derived antigen showed seroreactivity and was effective in stimulating the proliferation of mononuclear cells from seropositive individuals in vitro. Additionally, when appropriately adjuvanted, the anchor-less SAG1 successfully conferred protection to 60% of mice against a lethal challenge with *T. gondii* tachyzoites. Interestingly, when Letourneur et al. [102] utilized *P. pastoris* for the production of a SAG1 truncated protein lacking the C-terminal GPI-anchor with the single possible N-glycosylation site eliminated by site-directed mutagenesis, O-glycosylation occurred. The recombinant protein was secreted into medium as three variants of varying molecular masses due to the presence of O-linked oligosaccharides containing α1-2-, α1-3- or α1-6-linked mannoses. Moreover, it was shown that the percentage of glycosylated SAG1 proteins was dependent on the composition of yeast culture medium. The hyper-O-mannosylation of SAG1 did not seem to have any major effect on the conformation, as the recombinant antigen was recognized by specific anti-*T. gondii* antibodies in human sera. However, ELISA and Western blot assays indicated the reactivity of human sera against oligomannosidic epitopes present on the O-carbohydrates introduced by *P. pastoris*, which compromises the diagnostic utility of the recombinant SAG1 glycoprotein. These studies highlight that glycosylation in yeast systems does not reflect the native *T. gondii* protein structures. There is a potential for the host immune response to target yeast-derived glycosylation patterns or other yeast-specific protein modifications, which might complicate the use of such antigens in diagnostic or therapeutic applications. Moreover, atypical glycan structures introduced by *P. pastoris* are immunogenic and represent a potential limitation of this expression system. In practice, the choice of expression system often depends not only on the specific antigen being produced but also the selected amino acid fragment of that protein as well as the intended downstream application, requiring a balance between the advantages and limitations of yeast systems.

Other *T. gondii* antigens have also been successfully expressed in *P. pastoris* and validated according to their immunodiagnostic and/or immunoprophylactic utility [103,104,105,106,107,108,109,110]. However, their glycosylation pattern was not analyzed and discussed, which is crucial for the development of vaccines intended for human use. The U.S. Food and Drug Administration (FDA) has specific regulatory requirements for the glycan analysis of therapeutic proteins; this includes identifying the glycosylation sites on the protein, the structure of attached glycans, and the degree of occupancy of these sites [111]. These requirements are critical for ensuring the safety, efficacy, and quality of biopharmaceuticals, particularly because changes in glycosylation can significantly affect a drug’s biological function, immunogenicity, stability, and pharmacokinetics. This is especially challenging as glycosylation can be influenced by changes in the manufacturing process, such as growth conditions, and downstream processing [112]. In the European Union (EU), the regulatory framework for the glycan analysis of biopharmaceuticals has similar requirements and is primarily overseen by the European Medicines Agency (EMA) [113].

Studies predominantly report the use of a wild-type X-33 strain and pPICZ expression vectors. In these cases, recombinant protein expression is driven by the alcohol oxidase 1 promoter (P_AOX1_), which is unique in its regulation, being specifically induced by methanol and repressed by other common carbon sources such as glucose, glycerol, and ethanol [114]. This inducible and repressible system allows for a two-phase approach to fermentation: the growth phase is typically initiated with a repressing carbon source like glycerol, which promotes cell growth without triggering recombinant protein expression, while the induction phase is initiated by switching the carbon source to methanol, which not only provides energy but also induces the P_AOX1_ promoter to start the production of heterologous protein [115]. The main drawback of this approach is the need for the consistent, strict control of methanol concentration, which is often achieved by continuous feeding. The concentration of methanol must be high enough to effectively induce expression but not so high as to cause toxicity or metabolic stress to the cells [116]. Most protocols call for a final concentration of 0.5% to induce expression [117]; however, Lau et al. [105] found a methanol feeding of 0.75% to be optimal for GRA4 protein expression, while another study determined that a concentration of 1% was optimal for yeast growth and expression of the *Toxoplasma* ROP2 antigen [107]. These findings lead to the conclusion that methanol concentration should be optimized for each protein in order to obtain the highest possible yield. All reviewed studies based on the P_AOX1_ promoter report the use of standard pH 6 buffered complex medium containing glycerol (BMGY) during the growth phase and buffered complex medium containing methanol (BMMY) medium post-induction [103,104,105,106,110]. Efforts have been made to avoid the use of methanol by developing alternative promoters, such as the glyceraldehyde-3-phosphate dehydrogenase promoter (P_GAP_), which has been employed for the production of a chimeric SAG1-GRA2 antigen in *P. pastoris* strain GS115 in YPD medium [118].

While yeast expression systems offer advantages, such as the ability to perform eukaryotic-like PTMs, *E. coli* is still a significantly more popular choice for the production of *T. gondii* recombinant antigens.

## 5. Leishmania Tarentolae

It is thought that species most closely related may exhibit the highest potential to produce heterologous recombinant proteins. The presence of similar structures and mechanisms may show common yet unknown processes essential for the production of functional proteins [119]. It is therefore rational to conclude the potential of other protozoan parasites as expression systems for the production of *Toxoplasma* antigens; among them, *Leishmania tarentolae (L. tarentolae)* has garnered the most attention.

*Leishmania* parasites are single-celled protozoan eukaryotes responsible for a wide range of diseases, which are collectively known as leishmaniases [120]. Recent studies have highlighted that *L. tarentolae*, a lizard-hosted, non-pathogenic species within the *Leishmania* genus, has emerged as a novel expression system. Its N-glycosylation pattern closely resembles that found in mammals, more so than the patterns observed in yeast and insect cells [121]. *L. tarentolae* can be cultured to high cell densities in inexpensive media supplemented with hemin and has a fairly short doubling time of about 5 h [120]. Moreover, heterologous proteins can be produced within the cell or secreted into the medium, facilitating straightforward purification of the recombinant product [98].

There are very few examples of recombinant *T. gondii* antigenic protein expression using this system. Fritsche et al. [122] showed the successful expression and purification of the SAG2 protein in *Leishmania*, as confirmed through Western blot with anti-His6-antibodies. However, no further investigations to confirm the protein’s antigenicity and immunogenicity were carried out. Recently, a study reported the use of *L. tarentolae* to produce a fusion protein composed of *Toxoplasma* surface antigen SAG1 and a multimeric protein complex (SAP2) of *Leishmania mexicana* (*L. mexicana*). The author claims that SAP2, which is secreted into the culture supernatant, can be used as a carrier for the SAG1 protein of *T. gondii* in order to produce a protein with multiple subunits suitable for immunization; however, the obtained yield was very low [123].

A published study in 2021 reported the construction of a vector-based multi-epitope vaccine, containing immunodominant fragments of *Toxoplasma* SAG1, ROP16, GRA12, MIC4 and M2AP proteins, in *L. tarentolae.* The authors showed a strong stimulation of humoral and cellular immunity in vaccinated mice as well as relatively enhanced resistance and prolonged survival rates following parasite challenge [124].

## 6. Conclusions

The selection of an appropriate expression system for the production of *T. gondii* proteins is a multifaceted decision that significantly influences the practical and scientific outcomes of antigen development for diagnostics and vaccines. Even though the overwhelming majority of studies report the use of *E. coli,* there is no single universal standardized approach to protein production. The majority of scientific literature reports the use of the pET plasmid system (Novagen); however, the multitude of available host strains, vectors, affinity tags and expression conditions all affect key aspects such as protein yield, solubility, folding, toxicity management, metabolic burden, antigenicity and scalability. The choice of cloning and expression strategy must therefore be considered on a case-by-case basis. Typical cloning strategies include restriction-ligation cloning, or more recently, ligation-independent cloning facilitated by vectors such as pET30-Ek/Lic. A novel approach was proposed in 2021 [125]: the production of *T. gondii* SAG2, GRA2 and SAG2-GRA2 in genome-edited *E. coli* by CRISPR-Cas9. The study showed the feasibility of this approach.

Nearly all reports based on the T7 system describe the production of histidine-tagged proteins. It is important to note that although one of the main advantages of polyhistidine tags is thought to be their lack of effect on immunogenicity, the literature is inconclusive in this regard. Furthermore, there is a clear lack of studies regarding their impact on recombinant *Toxoplasma* antigens. In contrast, it has been indicated that the impact of other fusion tags on antigen–antibody interactions is dependent on the localization of immunogenic epitopes, in particular their proximity to the added domains [51,84,88]. Other bacteria have shown promise as expression systems; however, despite their potential, there have not yet been studies reporting the production of *Toxoplasma* antigenic proteins using these systems. This gap highlights an intriguing prospect for future research.

Although utilizing a prokaryotic expression system is burdened by their inability to perform PTMs, glycosylation in yeast systems does not reflect the native *T. gondii* protein structures and may vary drastically depending on the protein fragment chosen for expression. Moreover, atypical glycan structures introduced by *P. pastoris* are immunogenic, which complicates the use of such antigens in diagnostic or therapeutic applications. On the other hand, antigenic preparations obtained in yeast cells are free from contaminants derived from bacterial cells, which reduces the likelihood of cross-reactions, as *E. coli* infection is prevalent in humans [126]. The use of inactivated whole yeast cells or antigen display at the yeast cell surface has been proposed for the development of oral veterinary vaccines [127]; however, this approach has not been studied for toxoplasmosis. The practical application of using closely related species, i.e., *L. tarentolae,* for the production of heterologous *Toxoplasma* proteins remains limited with few documented cases. Possibly, the antigenic similarity of protozoan parasites may be a disadvantage, causing significant cross-reactions. Some reports demonstrate *T. gondii* protein production in mammalian cells [77,128,129]; however, this approach is much more expensive, time consuming and results in a low protein yield. Nevertheless, this area still has not been studied extensively.

There is still a significant lack of knowledge on the applicability of recombinant *Toxoplasma* antigens produced in alternative expression systems. Bacteria other than *E. coli*, yeast other than *P. pastoris*, microalgae, insect cells, plant-based systems and *Tetrahymena thermophila* (*T. thermophila*) are just some examples of expression systems that could be investigated. Moreover, cell free systems are an increasingly popular alternative and offer prospects for future studies. Research directly comparing the diagnostic and immunoprophylactic efficacy of *T. gondii* proteins produced in different expression systems is insufficient. Such comparisons could reveal important differences in how these proteins perform in diagnostic assays and vaccines, leading to more informed decisions in the development of tools for combating *Toxoplasma* infections. Moreover, many research articles fail to report the yield and purity of obtained protein preparations, which further complicates a direct comparison across different expression systems.

The production of properly folded, soluble antigens can be aided by the use of bioinformatics. Web-based tools are used to assess the physicochemical properties of proteins, identify post-translational modification sites as well as the structural and functional impacts of these modifications, and predict allergenicity, antigenicity and specific B-cell and T-cell epitopes. Each expression system offers distinctive advantages and disadvantages, and every recombinant protein should be individually analyzed in order to optimize the production process. In practice, the choice of expression platform is at least partially dictated by the available equipment and expertise.

In summary, this paper highlights the need for a case-by-case evaluation of cloning and expression strategies, considering factors such as glycan structure and the localization of immunogenic epitopes. A more nuanced and tailored approach to antigen production, using bioinformatics tools and exploring less conventional expression systems, could ultimately lead to significant advancements in combating *Toxoplasma* infections.

## Figures and Tables

**Table 1:** *T. gondii* antigens produced in *E. coli* using pGEX expression vectors.

Plasmid Vector	*E. coli* Host Strain	Protein	Expression Conditions	Yield	Application		Results	Reference, Year
					Diagnostic	Vaccine		
pGEX-1N	JM105	GST-H4 GST-H11	−	−	+	−	Both antigens were highly specific for *T. gondii* antibodies. H4- and H11-ELISA can distinguish between acute and chronic phases of toxoplasmosis.	1991, [32]
pGEX-2T	JM101	P22^a^_27–172_	LB, 37 °C, 3 h	−	+	−	Acute infection sera showed stronger IgG reaction with P22 protein (immunoblots and ELISA). IgA and IgM P22-ELISA showed no reactivity.	1992, [33]
pGEX-3X	SURE	GST-GRA6	−	−	+	−	GRA6-IgG EIA can distinguish between acute and chronic phases of toxoplasmosis.	1998, [34]
pGEX-3X pGEX2T pGEX-3X	JM101	GST-GRA1 GST-GRA6-Nt GST-GRA6-Ct	LB, 37 °C	−	+	−	GRA6-Ct IgG ELISA—10% sensitivity. GRA6-Nt IgG ELISA—96% sensitivity. GRA1 IgG ELISA—68% sensitivity	2000, [35]
pGEX-6p-1	BL21 Star (DE3)	SAG1/2, SAG1 SAG2	37 °C, 4 h	−	−	+	Vaccination with SAG1/2 protected 73% (11/15) of mice from a lethal challenge. SAG1 immunization—60% survival rate. SAG2 immunization—53% survival rate.	2004, [37]
pGEX-SN	−	GST-EC2 (MIC2_157–235_-MIC3_234–307_-SAG1_182–312_) GST-EC3 (GRA3_36–134_-GRA7_24–102_-M2AP_37–263_)	−	GST-EC2 8 mg/L GST-EC3 5 mg/L induced bacterial culture	+	−	IgG and IgM ELISAs using EC2 and EC3 perform similarly to commercial assays. IgM-capture assays with chimeric antigens enhance postnatal congenital toxoplasmosis diagnosis.	2006, [38]
pGEX-4T-1	BL21 (DE3)	GST-GRA2-SAG1A	−	−	+	−	GRA2-SAG1A rapid diagnostic test showed 100% specificity 100% and 97.1% sensitivity.	2013, [39]
pGEX-6P-1	Rosetta (DE3)	GST-ROP17	−	−	−	+	Intranasal ROP17 immunization in mice induces systemic and local immune responses. Provides protection against lethal *T. gondii* infections. Reduces tachyzoite burdens in host tissues. Increases animal survival rates.	2014, [36]
pGEX-4T-1	BL21 (DE3) pLysS	GST-GRA2 (aa 25–185, 25–135, 25–105, 75–185, 106–185, 25–105) GST-GRA3 (aa 39–138, 39–222) GST-ROP2 (aa 29–561, 29–323 324–561, 29–197, 324–483, 403–561, 324–430, 431–561) GST-MIC2 (aa 1–723, 1–651, 1–425, 219–651, 1–284, 142–425, 421–651, 1–215, 216–425, 142–284) GST-GRA2_31–71_-MIC2_1–284_	30 °C	−	+	−	GRA_231–71_-MIC_21–284_ shows a high diagnostic potential and may be used for developing a serological assay.	2014, [40]
pGEX-6p-1	BL21	GST-GRA7	LB, 37 °C, 6–8 h	−	+	−	GRA7-ELISA showed 92% sensitivity and 94% specificity. Results align closely with LAMP technique results.	2016, [41]
pGEX-6p-1	BL21	GST-GRA5	LB, 37 °C, 4 h	−	+	−	GRA5-ELISA with sera from hemodialysis patients showed 96% sensitivity and 93% specificity.	2021, [42]
pGEX-4T1	BL21 (DE3)	GRA15	LB, 37 °C, 4 h	−	−	+	GRA15 immunization in mice induced IgG1 and IgG2c, boosted spleen cell proliferation and interferon γ (IFN-γ) production, improved survival rates, and reduced parasite burden against the Pru strain.	2024, [43]

^a^ P22—SAG2.

## Data Availability

No new data was created or analyzed in this study. Data sharing is not applicable to this article.
